# Robot-assisted anterior transpedicular screw fixation with 3D printed implant for multiple cervical fractures: A case report

**DOI:** 10.1097/MD.0000000000031876

**Published:** 2022-12-09

**Authors:** Lei Pei, Wei Yuan, Xinchun Liu, Lin Cong, Yue Zhu

**Affiliations:** a Department of Orthopedics, First Hospital of China Medical University, Shenyang, China.

**Keywords:** 3D printed implant, anterior transpedicular screw, cervical fracture, fixation, robot-assisted surgery

## Abstract

The anterior transpedicular screw (ATPS) fixation in the cervical spine provides the advantages of both anterior and posterior cervical surgery; however, it poses a high risk of screw insertion. In addition, a 3D printed implant can match ATPS fixation and reconstruction of the vertebral body. Robot-assisted surgery can make this process easier and potentially improve the safety and accuracy of the procedure.

**Patient concerns:**

A 64-year-old female was hit by a heavy object 4 days before presentation to our hospital. The patient exhibited a muscle strength of 0/5 in both the lower limbs and 3/5 in both the upper limbs. The visual analogue scale (VAS) for the neck was 5 points. Computed tomography (CT) of the cervical spine identified a burst fracture of the C5 vertebral body, and longitudinal splitting fracture of the C6 and C7 vertebral bodies accompanied with a split in the lamina. Magnetic resonance imaging (MRI) revealed a spinal cord edema from the C3 to the C7 level.

**Diagnosis:**

Multiple cervical fractures with spinal cord injury.

**Interventions:**

Anterior C4-5 and C5-6 disc resection, C5 corpectomy, robot-assisted ATPS fixation with the 3-D printed implant was performed.

**Outcomes:**

The CT scans revealed a satisfactory location of the internal implantation without any signs of complications associated with implantations. Six months later, the muscle strength of both the upper limbs increased from level 3 to level 5, VAS of neck decreased from 5 to 0.

**Lessons:**

Robot-assisted ATPS internal fixation combined with custom implantation surgery using a 3D printed vertebral body provides an important solution to solve special cases.

## 1. Introduction

Multiple cervical fracture is a serious cervical spine injury, which often leads to spinal cord injury, quadriplegia, and may also become life-threatening in severe cases. The surgical treatment for subaxial cervical bursting fractures includes the anterior approach alone and anterior‐posterior approaches.^[1]^ The presently available anterior cervical spine implants do not offer high three-column instabilities for stronger fixation. Hence, the anterior‐posterior approach is used more commonly.^[2,3]^ Surgery using the anterior approach only is suitable for short-segment fractures. The biomechanical stability provided by traditional anterior plating for the treatment of severe three-column injury of the cervical spine, anterior multi-segment decompression and reconstruction, and osteoporosis is limited and insufficient.^[4]^ If anterior cervical implants can provide effective three-column stabilization, patients can avoid a longer operative time and greater risk of complications associated with the additional posterior approach.

In view of the aforementioned deficiencies of the traditional approaches, anterior transpedicular screw (ATPS) in the cervical spine has been studied extensively.^[5,6]^ Anatomical and morphological studies have further demonstrated the feasibility of ATPS insertion in the cervical spine.^[7–9]^ ATPS has the advantages of long screw length, good mechanical properties, firmness, and stability. With the development of surgical robot technology and medical 3D printed metal implants, conducting more personalized and precise surgeries has become possible. We present the first case of a robot-assisted ATPS fixation with a 3D printed implant and vertebral body reconstruction.

## 2. Case report

### 2.1. History

**Figure 1.**
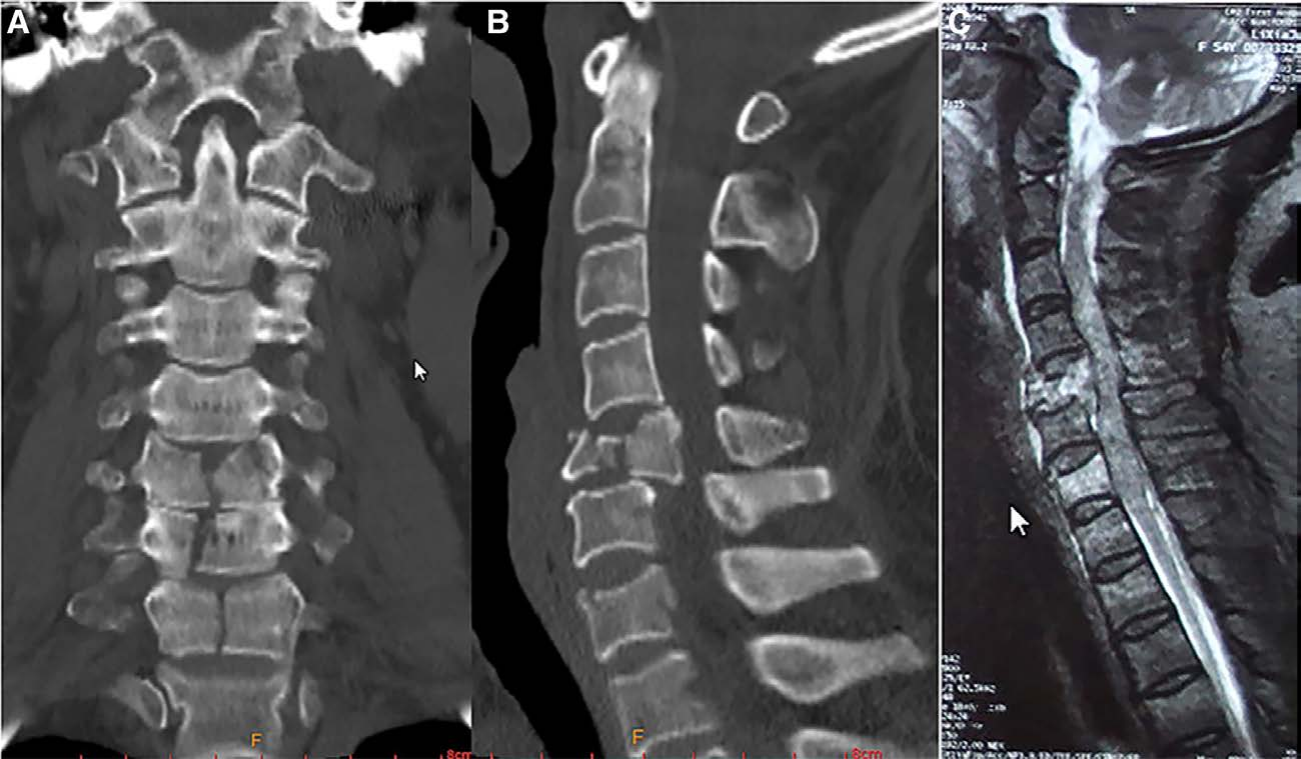
(a) Anteroposterior preoperative CT scan showing burst fracture with translational injury of C5 and longitudinal split fracture of C6 and C7, (b) lateral preoperative CT scan, and (c) lateral preoperative MRI showing a spinal cord edema extending from the epicenter of the injury up to C3 and down to C7.

A 64-year-old female was hit by a heavy object 4 days before presentation to our hospital. The patient exhibited a muscle strength of 0/5 in both the lower limbs and 3/5 in both the upper limbs. Hypoesthesia was found below the C6 plane. In addition, American Spinal Cord Injury Association impairment scale of A was observed. The score on the visual analogue scale (VAS) for the neck was 5 points. Computed tomography (CT) studies of the cervical spine identified a burst fracture of the C5 vertebral body, and longitudinal splitting fracture of the C6 and C7 vertebral bodies accompanied with a split in the lamina (Fig. 1a and b). The patient was then taken for an emergency cervical magnetic resonance imaging (MRI), which revealed a spinal cord edema from the C3 to the C7 level (Fig. 1c).

### 2.2. Preoperative design

**Figure 2.**
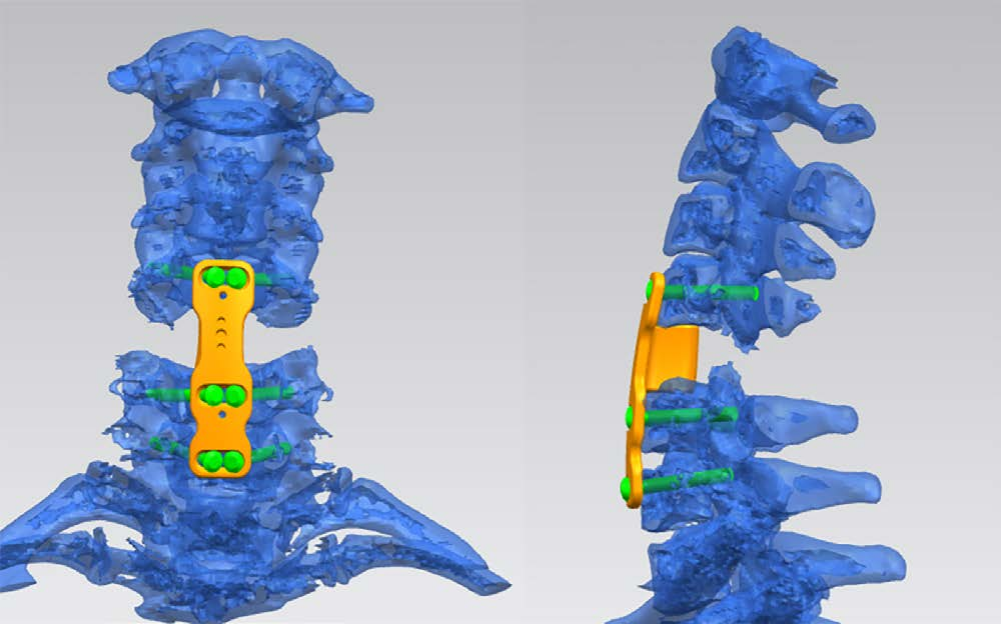
Preoperative renderings of the custom 3D-printed metal prosthesis.

Due to the burst fracture of C5 causing cervical instability and vertebral screws penetrating the fissures of the splitting fracture of C6 and C7, a conventional anterior cervical vertebral screw and plate fixation would not achieve firm fixation. ATPS fixation can achieve three-column fixation of the cervical spine, which is suitable for the treatment of this special case. Since the presently available and conventional anterior cervical plate cannot be matched with ATPS, a 3D printed metal implant with screw holes corresponding to ATPS, an artificial vertebral body, and an anterior cervical plate integration was designed before the operation according to the 3D-CT of the cervical spine (Fig. 2). To improve the accuracy of the screw placement, a robot-assisted ATPS technology surgery was planned.

### 2.3. Surgical procedure

**Figure 3.**
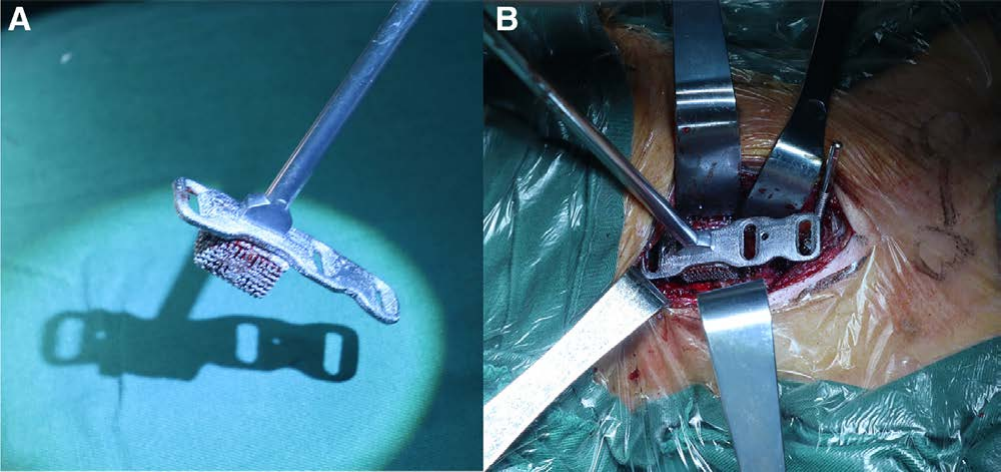
Intraoperative image of the 3D printed prosthesis implantation.

**Figure 4.**
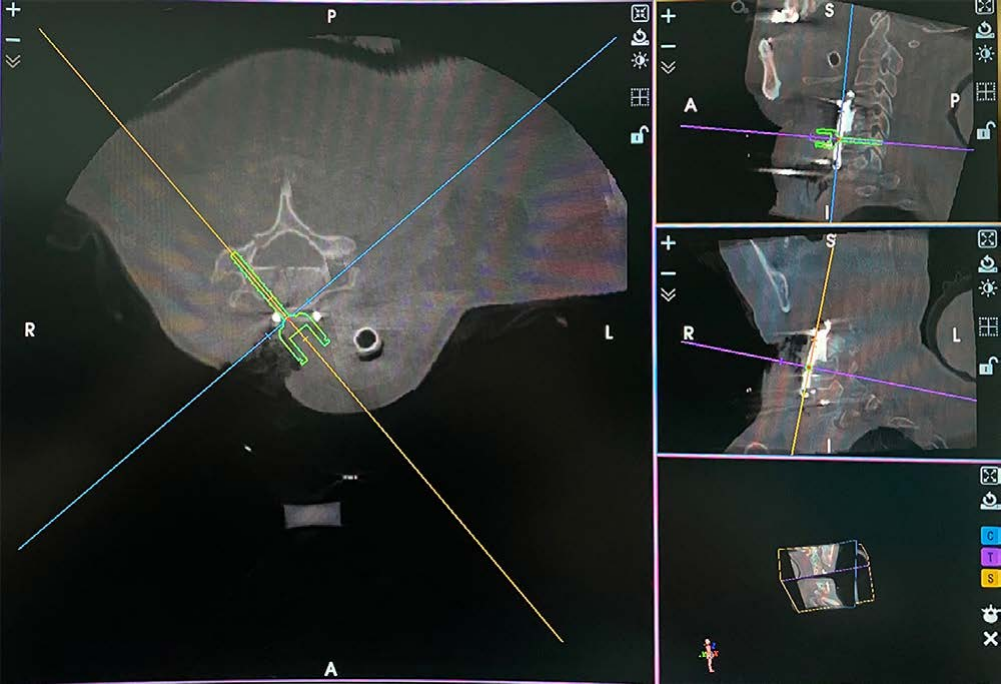
Intraoperative planning of pedicle screw inserting through the TiRobot system.

**Figure 5.**
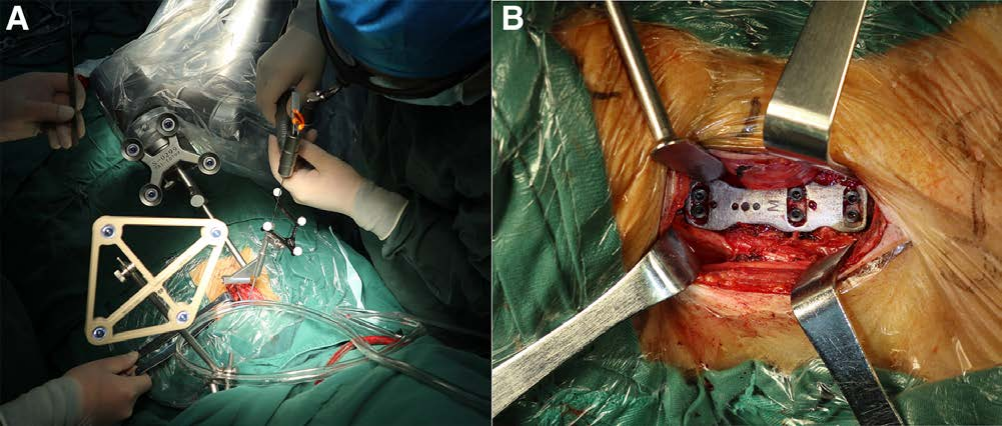
(a) Intraoperative image of K-wire inserting, and (b) inserting of the six anterior pedicle screws were completed.

**Figure 6.**
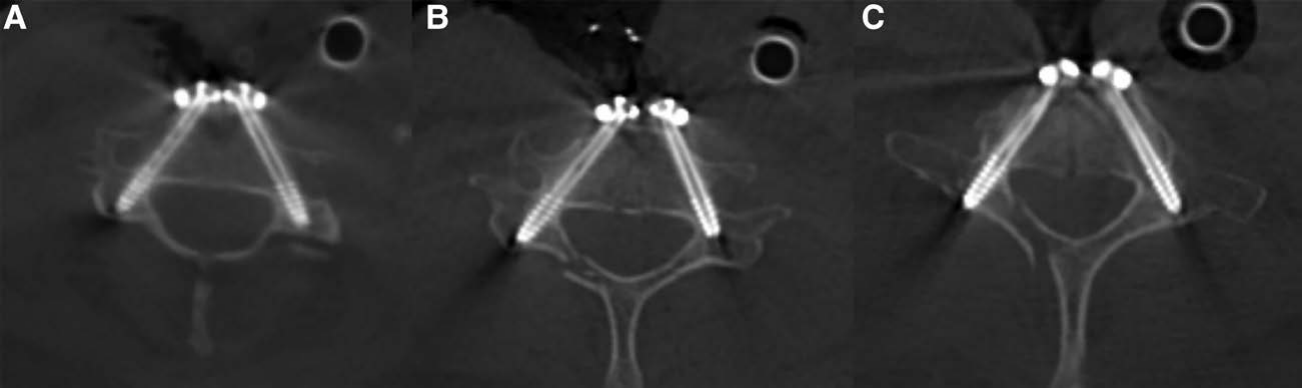
Intraoperative imaging of O-arm system verification (a) C4, (b) C6, and (c) C7.

**Figure 7.**
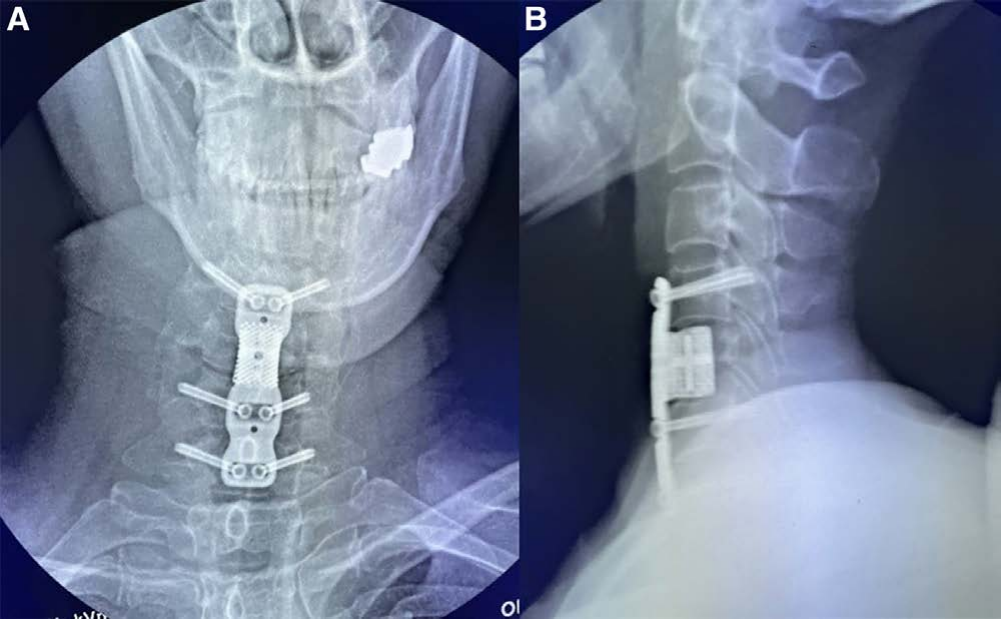
Intraoperative imaging of C-arm system verification (a) anterior scan, and (b) lateral scan.

The patient was positioned supine. Her head and neck were fixed with Mayfield tongs to keep the neck slightly flexed. We first performed an anterior C4-5 and C5-6 disc resection, C5 corpectomy, and fusion with the 3D printed prosthesis at C5 (Fig. 3). After the patient tracker was placed, a set of images were obtained by scanning with an intraoperative O-arm (Medtronic, US). The TiRobot system (TINAVI, Beijing, China) was used to complete the ATPS placement (Fig. 4). Through the cannula on the robotic arm, six bilateral pedicle screw guide wires (C4, C6, and C7) were inserted into the 3D printed plate screw holes (Fig. 5). The intraoperative O-arm scan verified the accuracy of the guide wires. The pedicle screws of appropriate lengths, ranging from 26 to 32 mm, were inserted according to the guide wires. The scan was repeated, which verified that all the six screws were located in the middle of the pedicle (Fig. 6). This was completely consistent with the ideal screw path designed before the operation. Finally, fluoroscopy image of cervical spine was taken by intraoperative C-arm (Fig. 7).

### 2.4. Follow-up

**Figure 8.**
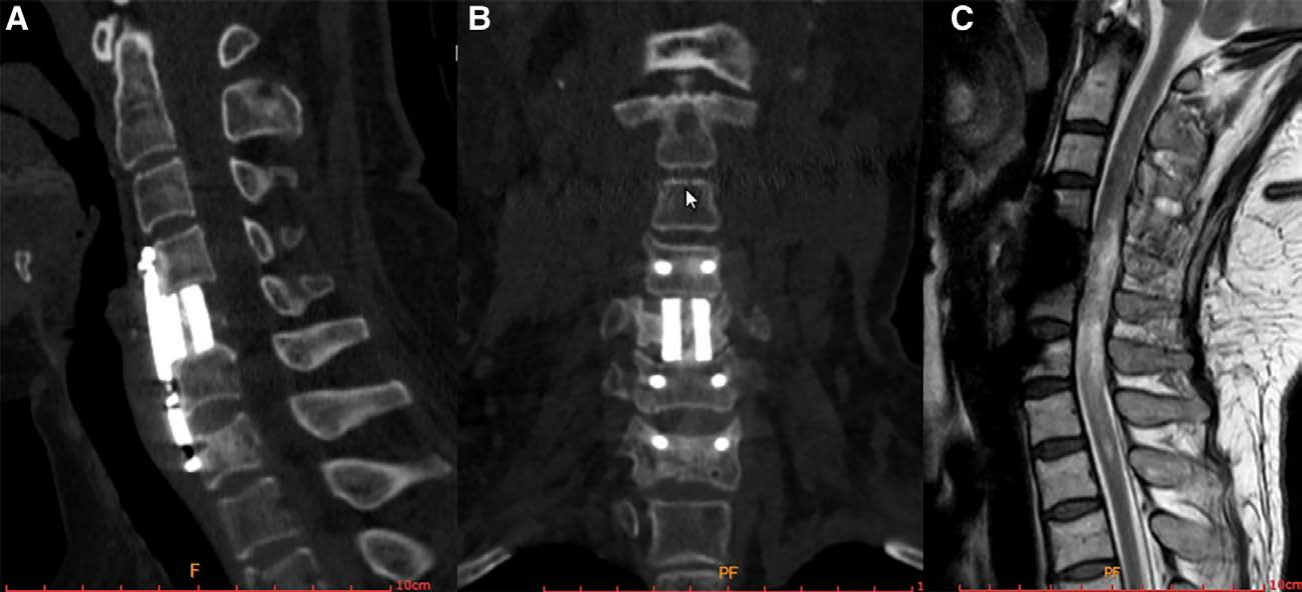
Post-operative CT and MRI scans at the six month of follow-up (a) lateral CT scan, (b) anterior CT scan, and (c) anterior MRI scan.

Postoperatively, a cervical hard collar was used to protect the neck of the patients for 6 weeks. The patient was transferred to the rehabilitation department of our hospital for further rehabilitation after 1 week. Six months later, the muscle strength of both the upper limbs increased from level 3 to level 5, VAS of neck decreased from 5 to 0, the accuracy of the pedicle screw placement was examined by CT scans and decompression was determined to be sufficient by reviewing the MR (Fig. 8). The results revealed a satisfactory location of the internal implantation without any signs of complications associated with implantations.

### 2.5. Patient consent statement

Written informed consent to participate in this study and publish of the case was provided by the patient and her legal guardian/next of kin. The approval for this study was obtained from the Institutional Review Board of the first hospital of China medical university (No. 2020-220-2).

## 3. Discussion

Traditional anterior cervical spine decompression and internal fixation mostly use vertebral screws, but are not suitable for cervical spine multi-segment injuries, severe osteoporosis, etc. Complications such as failure of bone graft fusion, false joint formation, loosening of internal fixation, etc may occur, which affects the efficacy of the surgery.^[10,11]^ When an internal fixation using a simple anterior cervical plate screw cannot be stabilized, it must be combined with posterior lateral mass screws or pedicle screws to assist internal fixation. In 2008, Koller et al proposed and investigated a new system of ATPS fixation technology to solve this problem.^[5–7]^ Compared with traditional anterior and posterior surgery, the ATPS fixation method combines the advantages of anterior and posterior cervical surgery, that is, it can treat cervical lesions with the anterior approach, while providing excellent stability of fixation of the pedicle screws.^[12,13]^ For the present case, a subtotal resection of the C5 vertebral bodies was decided as the surgical course. The C5, C6, and C7 vertebrae were fixed; however, a conventional anterior cervical vertebral screw and plate fixation would not achieve firm fixation. The main concern of the surgical plan was to achieve reliable fixation in the cervical spine using the anterior approach alone and avoid the cost of posterior surgery. Vertebral screws will penetrate the fissures of the splitting fracture, so ATPS can achieve three-column fixation of the cervical spine, which is suitable for the treatment of this special case.

The follow-up research further confirmed the anatomical feasibility and biomechanical stability of ATPS fixation. The biomechanical characteristics in cadaver experiments has earlier demonstrated that the pull-out strength of ATPS was about 2.5 times that of a vertebral body screw.^[14]^ Further, the primary stability after 2-level corpectomy reconstruction was comparable to posterior systems and superior to anterior plates.^[8]^ However, previous studies also found that ATPS fixation has a high risk of screw insertion, with a possibility of catastrophic complications such as injury to the vertebral artery, spinal cord, and nerve roots.^[12]^ Therefore, the accuracy of screw placement is very important.

There are no observable bony marks on the front surface of the lower cervical vertebral body, which increases the difficulty of ATPS placement. Currently, pedicle axial X-ray fluoroscopy is applied in clinical settings with accuracy rates ranging from 94.1% (21/22) to 95.5% (16/17).^[12,15]^ However, this method requires high surgical experience and expert anatomical knowledge. Patton et al^[16]^ compared the insertion results of 54 ATPS in nine cervical spine specimens assisted by X-ray fluoroscopy and navigation devices respectively. The accuracy rate of insertion was 42.6% in the fluoroscopy group and 66.7% in the navigation group, and the difference between the two groups was statistically significant. In 2013, Fu et al^[17]^ proposed a method for a path guide plate produced by rapid prototyping technology to assist screws insertion in vitro with a 91.7% accuracy rate (44/48). The application of navigation technology has improved the accuracy of nail placement from 95% to 100% in in vitro tests.^[7,18]^

Robot-assisted surgery is being researched and applied worldwide.^[19]^ Surgery robot systems can compensate the limitations of human motor skills. They can provide an output of 3D-CT images for complete preoperative planning and have strong motion control, high operation accuracy, good repeatability, and stability. However, there is no report on the clinical application of robot-assisted ATPS fixation. In previous studies,^[12,15]^ the insertion point was located in the central or contralateral side for C3 to C5, and the insertion of contralateral ATPS on one level was found to be difficult. However, in our practice, bilateral ATPS insertion trajectories may be designed on one level by the assistance of the robot system, and not only along the pedicle axis with the fluoroscope-assisted imaging. Comparing other clinical reports on the application of unilateral ATPS fixation in anterior cervical discectomy or corpectomy fusion,^[9,12,15]^ the plate with bilateral ATPS in the present case provided stronger fixation. Further, the accuracy of the robot creates favorable conditions for a successful execution.

Conventional anterior cervical plates do not use pedicle screw holes. Due to the lack of distinctive instruments for ATPS fixation, AXIS and AO reconstruction plates have mostly been used as substitutes to date.^[12]^ Since ATPS fixation requires a large angle of insertion, conventional instruments cannot meet this requirement. Further, the protruding screw tail has the potential risk of esophageal complications. Therefore, distinctive ATPS fixation instruments are being developed. In addition, 3D printed artificial vertebral body have been reported, which have the theoretical benefit of improved load distribution, superior osseointegration, reduced stress shielding, and possibly a decreased risk of subsidence.^[20,21]^ For this study, we designed and applied a new type of custom 3D printed bilateral transpedicular plate with artificial vertebral body based on the preoperative 3D-CT of the patient and obtained excellent clinical outcomes.

## 4. Conclusion

The robot-assisted ATPS could be feasible, safe, and accurate for multiple cervical fractures. Robot-assisted ATPS internal fixation combined with custom implantation surgery using a 3D printed vertebral body provides an important solution to solve special cases. The successful implementation of this operation has established a reliable, robot-assisted ATPS placement process that has further broadened the scope of use of orthopedic surgical robots.

## Author contributions

LP: Writing—Original Draft, Resources. WY, XL, LC: Conceptualization, Methodology. YZ: Supervision, Writing—Review & Editing. The final manuscript was approved by all authors.

**Conceptualization:** Lei Pei, Lin Cong, Wei Yuan, Xinchun Liu.

**Data curation:** Lin Cong, Wei Yuan, Xinchun Liu.

**Resources:** Yue Zhu.

**Validation:** Lin Cong, Wei Yuan, Xinchun Liu.

**Writing – original draft:** Lei Pei.

**Writing – review &amp; editing:** Yue Zhu.

## References

[1] Bozkus H Ames CP Chamberlain RH. Biomechanical analysis of rigid stabilization techniques for three-column injury in the lower cervical spine. Spine (Phila Pa 1976). 2005;30:915–22. 15834336 10.1097/01.brs.0000158949.37281.d7

[2] Do Koh Y Lim TH Won You J. A biomechanical comparison of modern anterior and posterior plate fixation of the cervical spine. Spine (Phila Pa 1976). 2001;26:15–21. 11148640 10.1097/00007632-200101010-00005

[3] Daubs MD. Early failures following cervical corpectomy reconstruction with titanium mesh cages and anterior plating. Spine (Phila Pa 1976). 2005;30:1402–6. 15959369 10.1097/01.brs.0000166526.78058.3c

[4] Koller H Hempfing A Ferraris L. 4- and 5-Level anterior fusions of the cervical spine: review of literature and clinical results. Eur Spine J. 2007;16:2055–71. 17605052 10.1007/s00586-007-0398-7 PMC2140121

[5] Koller H Hempfing A Acosta F. Cervical anterior transpedicular screw fixation. Part I: study on morphological feasibility, indications, and technical prerequisites. Eur Spine J. 2008;17:523–38. 18224358 10.1007/s00586-007-0572-y PMC2295270

[6] Koller H Acosta F Tauber M. Cervical anterior transpedicular screw fixation (ATPS)--Part II. Accuracy of manual insertion and pull-out strength of ATPS. Eur Spine J. 2008;17:539–55. 18224357 10.1007/s00586-007-0573-x PMC2295271

[7] Koller H Hitzl W Acosta F. In vitro study of accuracy of cervical pedicle screw insertion using an electronic conductivity device (ATPS part III). Eur Spine J. 2009;18:1300–13. 19575244 10.1007/s00586-009-1054-1 PMC2899545

[8] Koller H Schmidt R Mayer M. The stabilizing potential of anterior, posterior and combined techniques for the reconstruction of a 2-level cervical corpectomy model: biomechanical study and first results of ATPS prototyping. Eur Spine J. 2010;19:2137–48. 20589516 10.1007/s00586-010-1503-x PMC2997200

[9] Zhao L Li G Liu J. Radiological studies on the best entry point and trajectory of anterior cervical pedicle screw in the lower cervical spine. Eur Spine J. 2014;23:2175–81. 25056398 10.1007/s00586-014-3473-x

[10] McClelland S 3rd Passias PG Errico TJ. Inpatient versus outpatient anterior cervical discectomy and fusion: a perioperative complication analysis of 259,414 patients from the healthcare cost and utilization project databases. Int J Spine Surg. 2017;11:11. 28765795 10.14444/4011 PMC5537979

[11] Gruskay JA Fu M Basques BA. Factors affecting length of stay and complications after elective anterior cervical discectomy and fusion: a study of 2164 patients from the American college of surgeons national surgical quality improvement project database (ACS NSQIP). Clin Spine Surg. 2016;29:E34–42. 24525748 10.1097/BSD.0000000000000080

[12] Yukawa Y Kato F Ito K. Anterior cervical pedicle screw and plate fixation using fluoroscope-assisted pedicle axis view imaging: a preliminary report of a new cervical reconstruction technique. Eur Spine J. 2009;18:911–6. 19343377 10.1007/s00586-009-0949-1 PMC2899659

[13] Zhang Z Mu Z Zheng W. Anterior pedicle screw and plate fixation for cervical facet dislocation: case series and technical note. Spine J. 2016;16:123–9. 26409419 10.1016/j.spinee.2015.09.040

[14] Koktekir E Toktas ZO Seker A. Anterior transpedicular screw fixation of cervical spine: Is it safe? Morphological feasibility, technical properties, and accuracy of manual insertion. J Neurosurg Spine. 2015;22:596–604. 25815805 10.3171/2014.10.SPINE14669

[15] Aramomi M Masaki Y Koshizuka S. Anterior pedicle screw fixation for multilevel cervical corpectomy and spinal fusion. Acta Neurochir (Wien). 2008;150:575–82. discussion 82. 18431528 10.1007/s00701-008-1574-1

[16] Patton AG Morris RP Kuo YF. Accuracy of fluoroscopy versus computer-assisted navigation for the placement of anterior cervical pedicle screws. Spine (Phila Pa 1976). 2015;40:E404–10. 25599290 10.1097/BRS.0000000000000786

[17] Fu M Lin L Kong X. Construction and accuracy assessment of patient-specific biocompatible drill template for cervical anterior transpedicular screw (ATPS) insertion: an in vitro study. PLoS One. 2013;8:e53580. 23326461 10.1371/journal.pone.0053580 PMC3542371

[18] Bredow J Meyer C Scheyerer MJ. Accuracy of 3D fluoroscopy-navigated anterior transpedicular screw insertion in the cervical spine: an experimental study. Eur Spine J. 2016;25:1683–9. 26810977 10.1007/s00586-016-4403-x

[19] Bertelsen A Melo J Sanchez E. A review of surgical robots for spinal interventions. Int J Med Robot. 2013;9:407–22. 23239581 10.1002/rcs.1469

[20] Hunn SAM Koefman AJ Hunn AWM. 3D-printed titanium prosthetic reconstruction of the C2 vertebra: techniques and outcomes of three consecutive cases. Spine (Phila Pa 1976). 2020;45:667–72. 31809469 10.1097/BRS.0000000000003360

[21] Li X Wang Y Zhao Y. Multilevel 3D printing implant for reconstructing cervical spine with metastatic papillary thyroid carcinoma. Spine (Phila Pa 1976). 2017;42:E1326–30. 28498291 10.1097/BRS.0000000000002229

